# The growing significance of *delirium* in children

**DOI:** 10.62675/2965-2774.20250117

**Published:** 2025-12-08

**Authors:** Roberta Esteves Vieira de Castro, Yu Kawai, Alexandria Barry, Dickey Catherine Fuchs, Elizabeth Engstrom, Kristina A. Betters, Heidi A. B. Smith

**Affiliations:** 1 Universidade do Estado do Rio de Janeiro Hospital Universitário Pedro Ernesto Department of Pediatrics Rio de Janeiro RJ Brazil Department of Pediatrics, Hospital Universitário Pedro Ernesto, Universidade do Estado do Rio de Janeiro - Rio de Janeiro (RJ), Brazil.; 2 Instituto D’Or de Pesquisa e Ensino Department of Pediatrics Rio de Janeiro RJ Brazil Department of Pediatrics, Instituto D’Or de Pesquisa e Ensino - Rio de Janeiro (RJ), Brazil.; 3 Department of Pediatrics, Division of Pediatric Critical Care Rochester Minnesota United States Department of Pediatrics, Division of Pediatric Critical Care, Mayo Clinic Children's - Rochester, Minnesota, United States.; 4 Vanderbilt University Medical Center Department of Division of Allergy, Pulmonary and Critical Care Medicine, Center for Critical Illness, Brain Dysfunction and Survivorship Nashville Tennessee United States Department of Division of Allergy, Pulmonary and Critical Care Medicine, Center for Critical Illness, Brain Dysfunction and Survivorship, Vanderbilt University Medical Center - Nashville, Tennessee, United States.; 5 Vanderbilt University Medical Center Department of Pediatrics Department of Psychiatry and Behavioral Science, Division of Child and Adolescent Psychiatry Nashville Tennessee United States Department of Psychiatry and Behavioral Science, Division of Child and Adolescent Psychiatry, and Department of Pediatrics, Vanderbilt University Medical Center - Nashville, Tennessee, United States.; 6 Vanderbilt University Medical Center Department of Pediatrics Nashville Tennessee United States Department of Pediatrics, Vanderbilt University Medical Center - Nashville, Tennessee, United States.; 7 Vanderbilt University Medical Center Department of Anesthesiology Nashville Tennessee United States Department of Anesthesiology, Vanderbilt University Medical Center - Nashville, Tennessee, United States.

*Delirium* is a neuropsychiatric syndrome marked by acute disturbances in attention and awareness, often accompanied by cognitive impairment. Its severity typically fluctuates and arises as a direct physiological response to a medical or surgical condition.^(1–3)^ Although it has been extensively studied as a well-established entity in adult critical care, its recognition and management in neonatal/pediatric patients remain relatively nascent and have lagged due to a lack of standardized diagnostic tools in languages other than English and limited awareness among caregivers.^(4)^ Historically, the perception that children cannot experience *delirium* the same way as adults has contributed to this delay in its awareness.^(5)^ However, in recent years, research advances have dispelled these misconceptions, and pediatric *delirium* (PD) has garnered increased attention as it is not only prevalent but also clinically relevant due to its significant implications for the short- and long-term outcomes of affected children.^(1,2)^

This viewpoint emphasizes the need to understand PD - its epidemiology, risk factors, and complications - while recognizing the role of pediatric specialty groups, such as mini-MINDS, in advancing knowledge and care in the field.

## UNDERSTANDING PEDIATRIC *DELIRIUM*

An expanding body of evidence indicates that PD is frequently underdiagnosed. However, recent studies report prevalence rates in pediatric intensive care units (ICUs) ranging from 4% to over 70%, varying according to the patient population and diagnostic criteria applied.^(5–8)^

**Figure 1:**
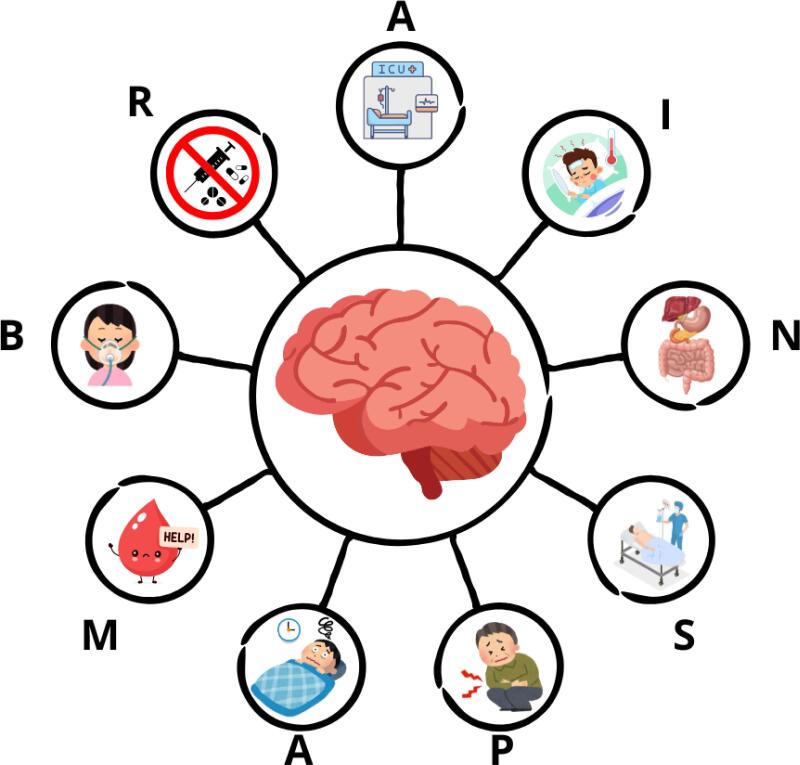
Brain maps.^(3,13)^

Pediatric *delirium* arises from a complex interplay of predisposing and precipitating factors, whose identification is crucial for prevention and early intervention.^(1,7)^ Key contributors include younger age, especially under 2 years, due to brain immaturity and dependence on caregivers; pre-existing neurological or developmental impairments; poor nutritional status; cyanotic heart disease; higher illness severity (e.g., elevated PRISM and PIM 3 scores); and prolonged hospitalization or mechanical ventilation (MV). Other factors involve exposure to sedatives (notably benzodiazepines), opioids, anticholinergics, steroids, antiepileptics, vasoactive drugs; deep sedation or coma; use of physical restraints; anemia and blood transfusions; absence of familiar caregivers; systemic infections and inflammation, which can affect the blood-brain barrier and neurochemistry; as well as sensory deprivation or overload in the pediatric ICU environment.^(1,2,5,7,9-12)^Figure 1 outlines key precipitating PD risk factors using the BRAIN MAPS acronym.^(13)^

**B - B**ring oxygen: anemia, decreased cardiac output, hypoxemia; **R - R**emove or **R**educe deliriogenic drugs: anticholinergics, benzodiazepines, opioids, steroids; **A - A**tmosphere: i.e. lights, noise, restraints, absent family, ‘strangers’, no schedule; **I - I**nfection, **I**mmobilization, **I**nflammation; **N - N**ew organ dysfunction; **M - M**etabolic disturbances: alkalosis, acidosis, hyponatremia, hypernatremia, hypokalemia, hyperkalemia, hypocalcemia, hypoglycemia; **A - A**wake: no bedtime routine, sleep-wake cycle disturbance; **P - P**ain: too much and not enough drug OR pain treated and now too much drug. **S – S**edation: assess need and set sedation target

Figure prepared in the Canva application

*Delirium* is classified into hypoactive, hyperactive, or mixed subtypes. Hypoactive cases, marked by low arousal, are more complex to recognize and are linked to worse outcomes. Hyperactive *delirium*, though more apparent due to agitation and emotional lability, is less common in pediatric ICUs.^(1)^ Using the Richmond Agitation and Sedation Scale (RASS), delirious children with a RASS score of zero to −3 are designated as having hypoactive *delirium*. *Delirium* is hyperactive when the RASS score is zero to +4.^(14)^ The mixed subtype presents features of both.^(1)^

Pediatric *delirium* consequences are profound, extending beyond the acute hospital stay. In the short term, PD is associated with prolonged MV, increased pediatric ICU and hospital length of stay, heightened mortality rates, and increased healthcare costs. The condition can exacerbate and can also be confused with pain and anxiety, further complicating recovery.^(1,2,7,9,11,12)^ Long-term evidence links PD to persistent cognitive deficits, emotional disturbances, higher hospital readmission rates, and reduced quality of life. *Delirium* during critical illness may contribute to pediatric post-intensive care syndrome (PICS), marked by lasting physical, cognitive, and psychological impairments. Pediatric *delirium* also impacts caregivers, as observing a child's altered mental state and clinical condition often causes significant parental stress and anxiety.^(15)^

## CHALLENGES AND OPPORTUNITIES IN PEDIATRIC *DELIRIUM* RESEARCH

Despite the growth in research into PD, the field still has many challenges to overcome. Unfortunately, difficulties in diagnosis remain a significant obstacle, as PD clinical manifestations often overlap with other conditions, such as anxiety, inadequately managed pain, iatrogenic withdrawal syndrome, or effects of over and under sedation. It is essential to highlight that these conditions can overlap with *delirium* and are relevant risk factors that are frequently missed.^(1,6)^ The development of pediatric-specific tools like the Preschool/Pediatric Confusion Assessment Method for the Intensive Care Unit (PEDs CAM-ICU series), the Cornell Assessment of Pediatric *Delirium* (CAP-D), and the Sophia Observation Withdrawal Symptoms - Pediatric *delirium* Scale (SOS-PD)^(1,2)^ represent a significant advance in PD early recognition. However, these tools still require wider implementation and validation in diverse clinical settings worldwide. In addition, research into the pathophysiology and neurobiological mechanisms underlying PD is urgently needed. Much remains to be unraveled about the interplay between critical illness, medications, and inflammation in the developing brain.^(7)^ As in adults, PD's complex and poorly understood pathophysiology hinders the development of targeted prevention and treatment strategies.^(16)^Table 1 outlines key research gaps in PD identified by the recent Peds PANDEM clinical practice guidelines from the Society of Critical Care Medicine (SCCM).^(1)^

**Table 1:** A framework for advancing pediatric *delirium* research^(1)^

Field	Focus areas
Antipsychotics	Antipsychotic medications’ role in preventing or treating *delirium* in neonatal ICU/pediatric ICU patients
Biomarkers	Identify potential biomarkers for diagnosing or predicting the outcomes of neonatal ICU/pediatric ICU *delirium*
Brain activity monitors	Assess whether brain activity monitoring is associated with the presence or severity of *delirium*
Bundled care practices	Evaluate the effect of bundled care strategies, such as the ABCDEF bundle, on neonatal ICU/pediatric ICU *delirium* and long-term patient outcomes
Dexmedetomidine	Additional research is needed to assess dexmedetomidine-based sedation and its relationship with *delirium* in critically ill children
Diagnosis	Need for development of tools for valid and reliable assessment of *delirium* in premature infants
Long-term outcomes	The connection between *delirium* and long-term consequences, including cognitive or executive impairment, psychological recovery, and PTSD
Opioid selection	The effect of opioid selection on the incidence or severity of *delirium*
Neonatal ICU and pediatric ICU environment	The influence of the neonatal ICU/pediatric ICU setting (including sleep quality, early mobilization, and dedicated family involvement) on the occurrence, intensity, and duration of *delirium*
Risk factors	There are still many gaps regarding risk factors for *delirium* in pediatrics, but there should be particular focus on infants under 6 months of age and patients with primary or secondary neurological injury
Sedation	The influence of sedation approach on the incidence and duration of *delirium*, considering both protocolized management and the selection of sedative agents (dexmedetomidine, ketamine, and barbiturates)
Sleep quality	Pharmacologic agents’ role to promote sleep quality and how this impacts *delirium*

ICU - intensive care unit; PTSD - post-traumatic stress disorder.

## THE ROLE OF MINI-MINDS

Our group, mini-MINDS (Maximizing ICU Recovery and miNimizing Brain Dysfunction in PediatricS), a pediatric special interest group under the American *Delirium* Society (ADS), has spearheaded initiatives to enhance awareness, education, and research on PD. It was created in 2021 and provides a platform for interdisciplinary collaboration, bringing together pediatricians, intensivists, neurologists, psychiatrists, psychologists, physiotherapists, child-life specialists, nurses, and researchers to tackle the complexities of PD. The group's contributions include supporting studies on PD epidemiology, risk factors, and outcomes; collaborating on evidence-based recommendations for PD prevention, diagnosis, and management; conducting workshops and educational sessions to improve PD recognition and understanding among healthcare providers worldwide; and emphasizing the importance of family involvement and minimizing environmental stressors in the pediatric ICU. Benefits that can be obtained from the group include instructing and encouraging the use of valid and reliable tools for PD diagnosis, avoiding its underdiagnosis, reducing the time of MV and the length of hospital stay, minimizing or avoiding long-term cognitive sequelae observed in patients who were hospitalized in the pediatric ICU even after hospital discharge, learning about the interaction of sedative medications and the brain as it impacts the mental status of the child, adopting actions to prevent *delirium* by all members of the interdisciplinary team in pediatric ICU, integrating researchers from all over the world, especially from North, Central and South America, disseminating knowledge about PD, and contributing to improving the quality of care.

Mini-MINDS envisions a strategic plan for the next 5 to 10 years to address key PD gaps. Priorities include promoting international studies on PD's impact, improving understanding of its neurobiology, and advancing age-appropriate screening tools in pediatric ICUs. The group also advocates for research on post-discharge outcomes, mentoring programs, and institutional protocols that support early detection and prevention, working toward a global, evidence-based standard of care through interdisciplinary collaboration.

Despite the growing recognition of PD, significant challenges remain in standardizing its screening and management in pediatric ICUs. In addition to technical barriers, cultural resistance to change hinders the adoption of validated protocols and tools. Thus, research is needed to better understand how to implement educational strategies, adapt workflows to the realities of each setting, and promote behavior changes that are sustained and strengthened across interdisciplinary teams. These challenges are greater in resource-limited settings, where overcoming entrenched practices is critical to improving early detection and long-term outcomes for children at risk for PD.

To learn more, visit: https://americandeliriumsociety.org/Pediatric-Special-Interest-Group

## CONCLUSION

Early pediatric ICUs were established with the primary goal of saving lives. Today, pediatric intensive care also prioritizes minimizing comorbidities and enhancing quality of life after discharge. Recognizing PD as an acute and urgent syndrome, rather than normal patient behavior, marked a pivotal shift in care. Daily, routine assessment of PD is critical in mitigating the short and long-term negative impact of *delirium* and to provide the most up-to-date evidence-driven medical care to the critically ill children.

## Data Availability

The contents underlying the research text are included in the manuscript.
